# Crystal structure of obscurine: a natural product isolated from the stem bark of *B. obscura*

**DOI:** 10.1107/S2056989015010567

**Published:** 2015-06-10

**Authors:** Bruno N. Lenta, Rodolphe J. Chouna, Beate Neumann, Hans-Georg Stammler, Norbert Sewald

**Affiliations:** a Department of Chemistry, Higher Teacher Training College, University of Yaoundé 1, PO Box 47, Yaoundé, Cameroon; b Department of Chemistry, University of Dschang, PO Box 371, Dschang, Cameroon; c Department of Chemistry, University of Bielefeld, PO Box 100131, 33501 Bielefeld, Germany

**Keywords:** crystal structure, obscurine, octa­hydro­naphthalene, benzodioxole, iso­butyl­acryl­amide, N—H⋯O hydrogen bonds

## Abstract

The title compound, C_24_H_31_NO_3_ {systematic name: (*E*)-3-[(1R*,2S*,4aS*,8aR*)-2-(benzo[*d*][1,3]dioxol-5-yl)-1,2,4a,5,6,7,8,8a-octa­hydro­naphthalen-1-yl]-*N*-iso­butyl­acryl­amide}, is a natural product isolated from the stem bark of *B. obscura*. It is composed of an octa­hydro­naphthalene ring system substituted with an essentially planar benzodioxole ring system [r.m.s. deviation = 0.012 Å] and an extended iso­butyl­acryl­amide group. In the crystal, mol­ecules are linked by N—H⋯O hydrogen bonds, forming chains propagating along [100]. The chains are linked by pairs of C—H⋯O hydrogen bonds, involving inversion-related benzodioxole ring systems, forming ribbons lying parallel to (010). There are also C—H⋯π inter­actions present within the ribbons.

## Related literature   

For background to the *Beilschmiedia* genus, medicinal plants used in Cameroon, see: Chouna *et al.* (2009 ▸, 2010 ▸, 2011 ▸); Lenta *et al.* (2009 ▸, 2011 ▸). For related structures, see: Balawsnt *et al.* (1975 ▸).
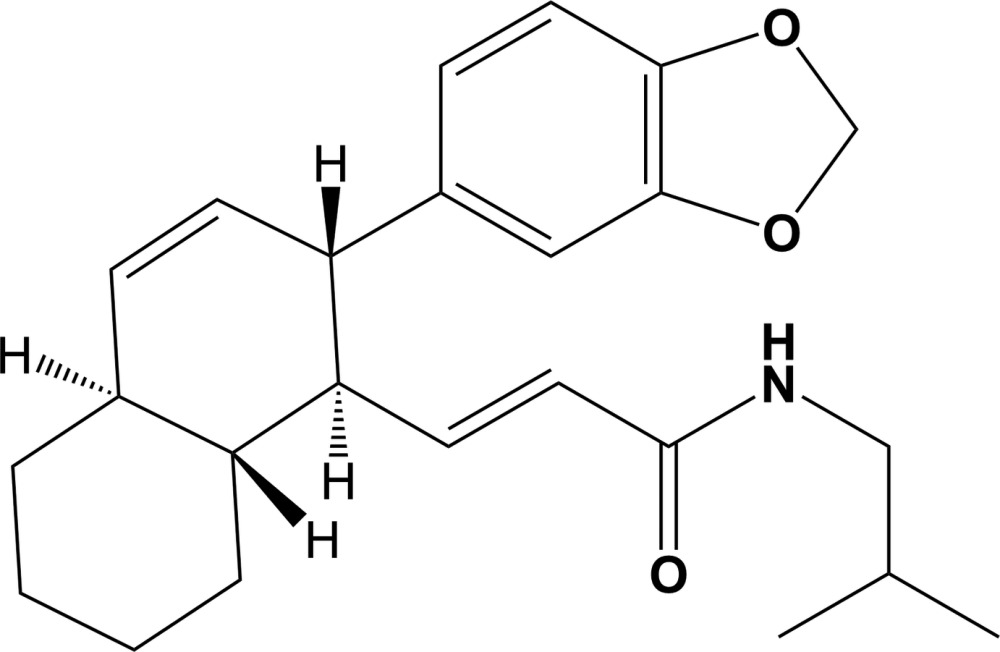


## Experimental   

### Crystal data  

C_24_H_31_NO_3_
*M* *_r_* = 381.50
Triclinic, 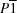

*a* = 5.14153 (16) Å
*b* = 9.7449 (3) Å
*c* = 20.4639 (5) Å
α = 98.839 (2)°
β = 90.946 (2)°
γ = 100.237 (3)°
*V* = 996.00 (5) Å^3^
*Z* = 2
Cu *K*α radiation
μ = 0.66 mm^−1^
*T* = 100 K
0.36 × 0.03 × 0.02 mm

### Data collection  

Agilent SuperNova, Dual, Cu at zero, Atlas diffractometer
Absorption correction: gaussian (*CrysAlis PRO*; Agilent, 2013 ▸) *T*_min_ = 0.798, *T*_max_ = 1.000
13324 measured reflections
3892 independent reflections
3425 reflections with *I* > 2σ(*I*)
*R*_int_ = 0.027

### Refinement  

*R*[*F* ^2^ > 2σ(*F* ^2^)] = 0.035
*wR*(*F* ^2^) = 0.091
*S* = 1.04
3892 reflections
377 parameters
All H-atom parameters refined
Δρ_max_ = 0.24 e Å^−3^
Δρ_min_ = −0.22 e Å^−3^

### 

Data collection: *CrysAlis PRO* (Agilent, 2013 ▸); cell refinement: *CrysAlis PRO*; data reduction: *CrysAlis PRO*; program(s) used to solve structure: *SHELXS97* (Sheldrick, 2008 ▸); program(s) used to refine structure: *SHELXL97* (Sheldrick, 2008 ▸); molecular graphics: *Mercury* (Macrae *et al.*, 2008 ▸); software used to prepare material for publication: *OLEX2* (Dolomanov *et al.*, 2009 ▸) and *PLATON* (Spek, 2009 ▸).

## Supplementary Material

Crystal structure: contains datablock(s) I, global. DOI: 10.1107/S2056989015010567/su5126sup1.cif

Structure factors: contains datablock(s) I. DOI: 10.1107/S2056989015010567/su5126Isup2.hkl

**Fig. 1.**
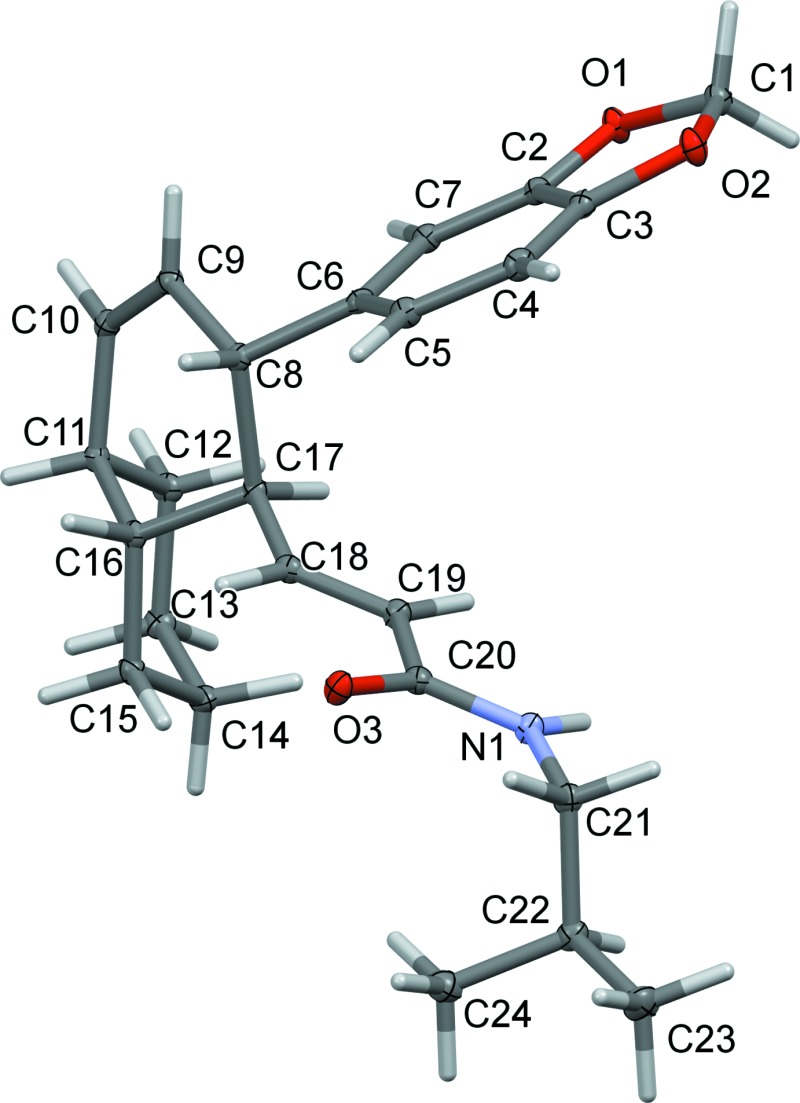
A view of the molecular structure of the title compound, with atom labelling. Displacement ellipsoids are drawn at the 50% probabilityl evel.

**Fig. 2.**
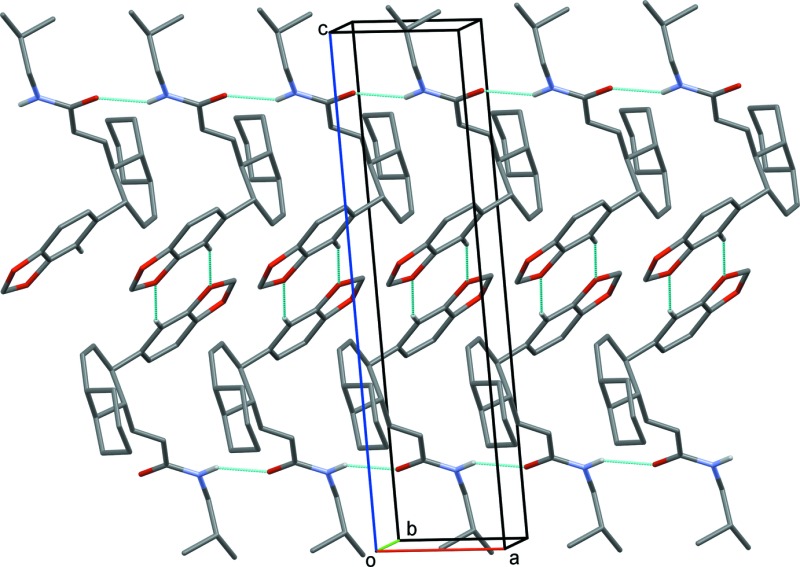
A view along the b axis of the crystal packing of the title compound. The hydrogen bonds are shown as dashed lines (see Table 1 for details).

. DOI: 10.1107/S2056989015010567/su5126fig1.tif
A view of the mol­ecular structure of the title compound, with atom labelling. Displacement ellipsoids are drawn at the 50% probabilityl evel.

b . DOI: 10.1107/S2056989015010567/su5126fig2.tif
A view along the *b* axis of the crystal packing of the title compound. The hydrogen bonds are shown as dashed lines (see Table 1 for details).

CCDC reference: 1404418

Additional supporting information:  crystallographic information; 3D view; checkCIF report

## Figures and Tables

**Table 1:** Hydrogen-bond geometry (, ) *Cg*2 is the centroid of the benzene ring C2C7.

*D*H*A*	*D*H	H*A*	*D* *A*	*D*H*A*
N1H1O3^i^	0.896(16)	2.105(16)	2.8938(13)	146.3(13)
C7H7O1^ii^	0.984(16)	2.503(16)	3.4264(15)	156.3(12)
C1H1*B* *Cg*2^i^	0.978(16)	2.595(15)	3.4578(12)	147.4(11)

Symmetry codes: (i) 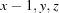; (ii) 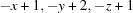.

## supplementary crystallographic information

### S1. Chemical context

Plants of the *Beilschmiedia* genus have been the subject of research inter­est (Lenta *et al.*, 2009, 2011; Chouna *et al.* 2011). Our inter­ests involve the isolation of active constituents from the stem bark and leaves of *B. obscura*, a medicinal plant used in Cameroon. Herein, we report on the crystal structure of the title compound, a natural product isolated from the stem bark of *B. obscura*.

### S2. Isolation and crystallization

The air-dried stem bark of *B. obscura* (400 g) was macerated with methanol at room temperature for 72 h. The methanol extract was concentrated under reduced pressure to give a residue of 16 g, which was selectively extracted with CH_2_Cl_2_ at room temperature to afford 4 g of the CH_2_Cl_2_ soluble residue. This extract was subjected to column chromatography (CC) over silica gel (0.023-0.20 mesh, Merck) and eluted with a gradient system of n-hexane/ CH_2_Cl_2_ and ( CH_2_Cl_2_/MeOH,) to afford obscurine (4.2 mg), the solid obtained was grounded and then recrystallised from a mixture of petroleum ether/di­chloro­metahne (1:1), yielding colourless needle-like crystals.

### S3. Refinement

Crystal data, data collection and structure refinement details are summarized in Table 2. All of the H atoms were all located in difference Fourier maps and freely refined.

### Crystal data

**Table:**

C_24_H_31_NO_3_	*Z* = 2
*M**_r_* = 381.50	*F*(000) = 412
Triclinic, *P*1	*D*_x_ = 1.272 Mg m^−^^3^
*a* = 5.14153 (16) Å	Cu *K*α radiation, λ = 1.5418 Å
*b* = 9.7449 (3) Å	Cell parameters from 7209 reflections
*c* = 20.4639 (5) Å	θ = 4.7–76.2°
α = 98.839 (2)°	µ = 0.66 mm^−^^1^
β = 90.946 (2)°	*T* = 100 K
γ = 100.237 (3)°	Needle, clear colourless
*V* = 996.00 (5) Å^3^	0.36 × 0.03 × 0.02 mm

### Data collection

**Table:**

Agilent SuperNova, Dual, Cu at zero, Atlas diffractometer	3892 independent reflections
Radiation source: SuperNova (Cu) X-ray Source	3425 reflections with *I* > 2σ(*I*)
Mirror monochromator	*R*_int_ = 0.027
Detector resolution: 5.3114 pixels mm^-1^	θ_max_ = 72.1°, θ_min_ = 4.4°
ω scans	*h* = −6→5
Absorption correction: gaussian (*CrysAlis PRO*; Agilent, 2013)	*k* = −11→12
*T*_min_ = 0.798, *T*_max_ = 1.000	*l* = −24→24
13324 measured reflections	

### Refinement

**Table:**

Refinement on *F*^2^	Primary atom site location: structure-invariant direct methods
Least-squares matrix: full	Hydrogen site location: difference Fourier map
*R*[*F*^2^ > 2σ(*F*^2^)] = 0.035	All H-atom parameters refined
*wR*(*F*^2^) = 0.091	*w* = 1/[σ^2^(*F*_o_^2^) + (0.0424*P*)^2^ + 0.3477*P*] where *P* = (*F*_o_^2^ + 2*F*_c_^2^)/3
*S* = 1.04	(Δ/σ)_max_ = 0.001
3892 reflections	Δρ_max_ = 0.24 e Å^−^^3^
377 parameters	Δρ_min_ = −0.22 e Å^−^^3^
0 restraints	

### Special details

**Table:**

Geometry. All e.s.d.'s (except the e.s.d. in the dihedral angle between two l.s. planes) are estimated using the full covariance matrix. The cell e.s.d.'s are taken into account individually in the estimation of e.s.d.'s in distances, angles and torsion angles; correlations between e.s.d.'s in cell parameters are only used when they are defined by crystal symmetry. An approximate (isotropic) treatment of cell e.s.d.'s is used for estimating e.s.d.'s involving l.s. planes.

### Fractional atomic coordinates and isotropic or equivalent isotropic displacement parameters (Å^2^)

**Table:**

	*x*	*y*	*z*	*U*_iso_*/*U*_eq_	
O3	0.99052 (16)	0.81354 (9)	0.86676 (4)	0.01947 (19)	
O1	0.32116 (17)	0.81350 (9)	0.50886 (4)	0.0198 (2)	
O2	0.26950 (17)	0.59230 (9)	0.54145 (4)	0.0211 (2)	
N1	0.5440 (2)	0.79089 (11)	0.87047 (5)	0.0177 (2)	
C7	0.7117 (2)	0.92262 (12)	0.58231 (6)	0.0155 (2)	
C6	0.8900 (2)	0.89637 (12)	0.62993 (6)	0.0149 (2)	
C14	0.9558 (2)	1.40385 (13)	0.81143 (6)	0.0189 (3)	
C18	0.9906 (2)	1.00932 (12)	0.77808 (6)	0.0161 (2)	
C19	0.7706 (2)	0.94294 (12)	0.80006 (6)	0.0172 (2)	
C4	0.6528 (2)	0.65272 (13)	0.62212 (6)	0.0183 (2)	
C16	1.2263 (2)	1.23586 (12)	0.74705 (6)	0.0152 (2)	
C17	1.0186 (2)	1.10091 (12)	0.72506 (6)	0.0147 (2)	
C2	0.5136 (2)	0.81316 (12)	0.55642 (6)	0.0154 (2)	
C22	0.4536 (2)	0.77083 (13)	0.98764 (6)	0.0195 (3)	
C8	1.1097 (2)	1.01511 (12)	0.66128 (6)	0.0150 (2)	
C3	0.4823 (2)	0.68151 (12)	0.57583 (6)	0.0162 (2)	
C5	0.8593 (2)	0.76299 (12)	0.64871 (6)	0.0174 (2)	
C20	0.7803 (2)	0.84434 (12)	0.84886 (6)	0.0161 (2)	
C21	0.5080 (2)	0.69546 (13)	0.91942 (6)	0.0182 (3)	
C12	1.0346 (2)	1.41782 (12)	0.69190 (6)	0.0174 (2)	
C13	1.0029 (3)	1.50233 (13)	0.75965 (6)	0.0192 (3)	
C9	1.2170 (2)	1.11024 (13)	0.61238 (6)	0.0163 (2)	
C11	1.2589 (2)	1.33271 (12)	0.69369 (6)	0.0153 (2)	
C10	1.2835 (2)	1.24957 (13)	0.62661 (6)	0.0166 (2)	
C23	0.3498 (3)	0.66495 (16)	1.03273 (7)	0.0293 (3)	
C15	1.1801 (2)	1.32087 (13)	0.81431 (6)	0.0178 (2)	
C1	0.1586 (2)	0.67567 (13)	0.49990 (6)	0.0186 (3)	
C24	0.6991 (3)	0.87158 (15)	1.01892 (7)	0.0260 (3)	
H16	1.402 (3)	1.2038 (15)	0.7515 (7)	0.016 (3)*	
H11	1.426 (3)	1.4037 (15)	0.7059 (7)	0.015 (3)*	
H17	0.844 (3)	1.1256 (15)	0.7138 (7)	0.015 (3)*	
H21A	0.670 (3)	0.6545 (15)	0.9203 (7)	0.019 (4)*	
H15A	1.345 (3)	1.3876 (15)	0.8301 (7)	0.017 (3)*	
H4	0.633 (3)	0.5592 (17)	0.6353 (8)	0.026 (4)*	
H9	1.238 (3)	1.0624 (15)	0.5679 (7)	0.018 (3)*	
H5	0.986 (3)	0.7444 (16)	0.6806 (8)	0.023 (4)*	
H12A	0.862 (3)	1.3498 (16)	0.6770 (7)	0.019 (4)*	
H18	1.159 (3)	0.9934 (15)	0.7974 (7)	0.020 (4)*	
H7	0.730 (3)	1.0156 (17)	0.5680 (7)	0.022 (4)*	
H13A	1.169 (3)	1.5731 (16)	0.7739 (8)	0.023 (4)*	
H14A	0.942 (3)	1.4596 (16)	0.8555 (8)	0.023 (4)*	
H21B	0.354 (3)	0.6183 (16)	0.9026 (7)	0.022 (4)*	
H1	0.399 (3)	0.8194 (16)	0.8567 (7)	0.022 (4)*	
H1A	0.160 (3)	0.6301 (16)	0.4528 (8)	0.023 (4)*	
H8	1.260 (3)	0.9720 (15)	0.6758 (7)	0.019 (4)*	
H12B	1.068 (3)	1.4823 (16)	0.6573 (8)	0.021 (4)*	
H14B	0.780 (3)	1.3357 (16)	0.7997 (8)	0.023 (4)*	
H1B	−0.020 (3)	0.6828 (15)	0.5137 (7)	0.021 (4)*	
H22	0.313 (3)	0.8255 (15)	0.9805 (7)	0.018 (3)*	
H10	1.350 (3)	1.3042 (16)	0.5915 (8)	0.023 (4)*	
H15B	1.146 (3)	1.2548 (17)	0.8489 (8)	0.025 (4)*	
H13B	0.851 (3)	1.5540 (17)	0.7578 (8)	0.026 (4)*	
H24A	0.841 (3)	0.8171 (19)	1.0287 (9)	0.036 (5)*	
H24B	0.660 (3)	0.9237 (19)	1.0614 (9)	0.037 (5)*	
H23A	0.483 (4)	0.609 (2)	1.0412 (9)	0.041 (5)*	
H19	0.599 (3)	0.9535 (17)	0.7834 (8)	0.029 (4)*	
H24C	0.775 (3)	0.9404 (19)	0.9890 (9)	0.036 (5)*	
H23B	0.304 (4)	0.717 (2)	1.0772 (10)	0.043 (5)*	
H23C	0.187 (4)	0.601 (2)	1.0128 (9)	0.043 (5)*	

### Atomic displacement parameters (Å^2^)

**Table:**

	*U*^11^	*U*^22^	*U*^33^	*U*^12^	*U*^13^	*U*^23^
O3	0.0158 (4)	0.0234 (4)	0.0221 (4)	0.0068 (3)	0.0012 (3)	0.0090 (3)
O1	0.0191 (4)	0.0170 (4)	0.0230 (4)	0.0005 (3)	−0.0059 (3)	0.0060 (3)
O2	0.0198 (4)	0.0153 (4)	0.0277 (5)	−0.0001 (3)	−0.0058 (4)	0.0058 (3)
N1	0.0154 (5)	0.0214 (5)	0.0187 (5)	0.0052 (4)	0.0010 (4)	0.0087 (4)
C7	0.0162 (6)	0.0148 (5)	0.0173 (6)	0.0050 (4)	0.0025 (4)	0.0048 (4)
C6	0.0153 (5)	0.0152 (5)	0.0152 (5)	0.0049 (4)	0.0034 (4)	0.0031 (4)
C14	0.0176 (6)	0.0216 (6)	0.0169 (6)	0.0042 (5)	0.0017 (5)	0.0007 (5)
C18	0.0175 (6)	0.0160 (5)	0.0157 (6)	0.0054 (4)	−0.0008 (4)	0.0029 (4)
C19	0.0161 (6)	0.0199 (6)	0.0176 (6)	0.0060 (5)	0.0000 (4)	0.0059 (4)
C4	0.0207 (6)	0.0143 (6)	0.0218 (6)	0.0047 (5)	0.0027 (5)	0.0069 (4)
C16	0.0134 (5)	0.0160 (5)	0.0171 (6)	0.0040 (4)	−0.0006 (4)	0.0038 (4)
C17	0.0134 (5)	0.0166 (6)	0.0154 (6)	0.0048 (4)	−0.0001 (4)	0.0041 (4)
C2	0.0153 (5)	0.0175 (6)	0.0150 (5)	0.0059 (4)	0.0018 (4)	0.0043 (4)
C22	0.0195 (6)	0.0213 (6)	0.0200 (6)	0.0066 (5)	0.0031 (5)	0.0070 (5)
C8	0.0138 (5)	0.0154 (5)	0.0170 (6)	0.0049 (4)	0.0006 (4)	0.0038 (4)
C3	0.0147 (5)	0.0146 (5)	0.0188 (6)	0.0020 (4)	0.0022 (4)	0.0019 (4)
C5	0.0187 (6)	0.0172 (6)	0.0185 (6)	0.0067 (5)	0.0006 (5)	0.0056 (4)
C20	0.0175 (6)	0.0158 (5)	0.0156 (6)	0.0043 (4)	0.0004 (4)	0.0027 (4)
C21	0.0194 (6)	0.0169 (6)	0.0193 (6)	0.0027 (5)	0.0008 (5)	0.0068 (4)
C12	0.0185 (6)	0.0172 (6)	0.0181 (6)	0.0060 (5)	−0.0004 (5)	0.0045 (4)
C13	0.0198 (6)	0.0172 (6)	0.0218 (6)	0.0070 (5)	0.0003 (5)	0.0022 (5)
C9	0.0141 (6)	0.0189 (6)	0.0164 (6)	0.0039 (4)	0.0017 (4)	0.0032 (4)
C11	0.0135 (5)	0.0154 (5)	0.0171 (6)	0.0025 (4)	0.0006 (4)	0.0033 (4)
C10	0.0145 (6)	0.0187 (6)	0.0176 (6)	0.0031 (4)	0.0021 (4)	0.0061 (4)
C23	0.0335 (8)	0.0297 (7)	0.0245 (7)	−0.0006 (6)	0.0050 (6)	0.0109 (6)
C15	0.0181 (6)	0.0186 (6)	0.0164 (6)	0.0026 (5)	−0.0014 (5)	0.0026 (4)
C1	0.0181 (6)	0.0167 (6)	0.0203 (6)	0.0012 (5)	−0.0015 (5)	0.0037 (4)
C24	0.0296 (7)	0.0235 (7)	0.0233 (7)	0.0028 (6)	−0.0010 (6)	0.0018 (5)

### Geometric parameters (Å, º)

**Table:**

O3—C20	1.2363 (15)	C2—C3	1.3838 (16)
O1—C2	1.3772 (14)	C22—C21	1.5289 (17)
O1—C1	1.4341 (15)	C22—C23	1.5213 (18)
O2—C3	1.3772 (14)	C22—C24	1.5189 (18)
O2—C1	1.4386 (15)	C22—H22	0.991 (15)
N1—C20	1.3443 (16)	C8—C9	1.5120 (16)
N1—C21	1.4606 (15)	C8—H8	1.006 (15)
N1—H1	0.894 (16)	C5—H5	0.973 (16)
C7—C6	1.4093 (16)	C21—H21A	0.987 (16)
C7—C2	1.3705 (17)	C21—H21B	1.004 (16)
C7—H7	0.984 (16)	C12—C13	1.5269 (16)
C6—C8	1.5199 (16)	C12—C11	1.5384 (16)
C6—C5	1.3945 (16)	C12—H12A	1.020 (15)
C14—C13	1.5300 (17)	C12—H12B	1.014 (16)
C14—C15	1.5270 (17)	C13—H13A	1.007 (16)
C14—H14A	0.988 (16)	C13—H13B	1.003 (17)
C14—H14B	1.021 (16)	C9—C10	1.3250 (17)
C18—C19	1.3216 (18)	C9—H9	0.973 (15)
C18—C17	1.5007 (16)	C11—C10	1.5024 (16)
C18—H18	0.994 (15)	C11—H11	1.006 (15)
C19—C20	1.4944 (16)	C10—H10	0.988 (16)
C19—H19	0.970 (17)	C23—H23A	0.98 (2)
C4—C3	1.3732 (17)	C23—H23B	1.025 (19)
C4—C5	1.4050 (17)	C23—H23C	0.99 (2)
C4—H4	0.978 (16)	C15—H15A	0.990 (15)
C16—C17	1.5411 (16)	C15—H15B	1.025 (16)
C16—C11	1.5421 (16)	C1—H1A	0.997 (16)
C16—C15	1.5385 (16)	C1—H1B	0.978 (16)
C16—H16	1.016 (15)	C24—H24A	1.010 (18)
C17—C8	1.5640 (16)	C24—H24B	0.980 (18)
C17—H17	1.002 (14)	C24—H24C	1.010 (19)
			
C2—O1—C1	106.11 (9)	C4—C5—H5	118.5 (9)
C3—O2—C1	105.76 (9)	O3—C20—N1	123.23 (11)
C20—N1—C21	124.03 (10)	O3—C20—C19	121.94 (11)
C20—N1—H1	119.1 (10)	N1—C20—C19	114.83 (10)
C21—N1—H1	116.7 (10)	N1—C21—C22	112.10 (10)
C6—C7—H7	121.2 (9)	N1—C21—H21A	106.5 (9)
C2—C7—C6	117.32 (11)	N1—C21—H21B	106.8 (9)
C2—C7—H7	121.5 (9)	C22—C21—H21A	112.0 (9)
C7—C6—C8	119.41 (10)	C22—C21—H21B	110.2 (9)
C5—C6—C7	119.69 (11)	H21A—C21—H21B	109.0 (12)
C5—C6—C8	120.89 (10)	C13—C12—C11	111.67 (10)
C13—C14—H14A	109.7 (9)	C13—C12—H12A	109.2 (8)
C13—C14—H14B	108.9 (9)	C13—C12—H12B	110.8 (9)
C15—C14—C13	111.11 (10)	C11—C12—H12A	108.9 (8)
C15—C14—H14A	109.2 (9)	C11—C12—H12B	110.0 (9)
C15—C14—H14B	109.8 (9)	H12A—C12—H12B	106.0 (12)
H14A—C14—H14B	108.0 (12)	C14—C13—H13A	107.7 (9)
C19—C18—C17	128.07 (11)	C14—C13—H13B	109.8 (9)
C19—C18—H18	116.3 (9)	C12—C13—C14	110.15 (10)
C17—C18—H18	115.5 (9)	C12—C13—H13A	110.0 (9)
C18—C19—C20	120.79 (11)	C12—C13—H13B	110.4 (9)
C18—C19—H19	120.9 (10)	H13A—C13—H13B	108.8 (12)
C20—C19—H19	118.3 (10)	C8—C9—H9	115.5 (9)
C3—C4—C5	116.89 (11)	C10—C9—C8	124.58 (11)
C3—C4—H4	121.4 (9)	C10—C9—H9	119.9 (9)
C5—C4—H4	121.7 (9)	C16—C11—H11	107.0 (8)
C17—C16—C11	111.83 (9)	C12—C11—C16	112.87 (10)
C17—C16—H16	106.3 (8)	C12—C11—H11	106.4 (8)
C11—C16—H16	106.0 (8)	C10—C11—C16	110.93 (9)
C15—C16—C17	114.84 (10)	C10—C11—C12	110.45 (10)
C15—C16—C11	110.08 (9)	C10—C11—H11	109.0 (8)
C15—C16—H16	107.2 (8)	C9—C10—C11	123.82 (11)
C18—C17—C16	110.54 (9)	C9—C10—H10	119.5 (9)
C18—C17—C8	108.26 (9)	C11—C10—H10	116.7 (9)
C18—C17—H17	110.5 (8)	C22—C23—H23A	110.8 (11)
C16—C17—C8	109.30 (9)	C22—C23—H23B	110.0 (11)
C16—C17—H17	110.5 (8)	C22—C23—H23C	110.8 (11)
C8—C17—H17	107.6 (8)	H23A—C23—H23B	108.3 (15)
O1—C2—C3	109.80 (10)	H23A—C23—H23C	109.1 (16)
C7—C2—O1	127.48 (10)	H23B—C23—H23C	107.8 (15)
C7—C2—C3	122.71 (11)	C14—C15—C16	113.29 (10)
C21—C22—H22	106.4 (8)	C14—C15—H15A	108.9 (8)
C23—C22—C21	110.96 (11)	C14—C15—H15B	110.5 (9)
C23—C22—H22	108.2 (9)	C16—C15—H15A	108.7 (8)
C24—C22—C21	111.12 (11)	C16—C15—H15B	109.8 (9)
C24—C22—C23	110.86 (11)	H15A—C15—H15B	105.3 (12)
C24—C22—H22	109.2 (9)	O1—C1—O2	108.07 (9)
C6—C8—C17	111.47 (9)	O1—C1—H1A	109.5 (9)
C6—C8—H8	108.3 (8)	O1—C1—H1B	109.9 (9)
C17—C8—H8	106.4 (8)	O2—C1—H1A	108.7 (9)
C9—C8—C6	111.81 (9)	O2—C1—H1B	108.4 (9)
C9—C8—C17	111.61 (9)	H1A—C1—H1B	112.2 (12)
C9—C8—H8	107.0 (9)	C22—C24—H24A	110.3 (10)
O2—C3—C2	110.17 (10)	C22—C24—H24B	110.8 (11)
C4—C3—O2	128.50 (11)	C22—C24—H24C	112.1 (10)
C4—C3—C2	121.32 (11)	H24A—C24—H24B	106.4 (14)
C6—C5—C4	122.06 (11)	H24A—C24—H24C	107.5 (14)
C6—C5—H5	119.5 (9)	H24B—C24—H24C	109.5 (14)
			
O1—C2—C3—O2	0.14 (14)	C3—O2—C1—O1	2.82 (12)
O1—C2—C3—C4	178.82 (11)	C3—C4—C5—C6	0.73 (18)
C7—C6—C8—C17	89.64 (12)	C5—C6—C8—C17	−88.97 (13)
C7—C6—C8—C9	−36.07 (14)	C5—C6—C8—C9	145.33 (11)
C7—C6—C5—C4	−0.73 (18)	C5—C4—C3—O2	178.46 (11)
C7—C2—C3—O2	−179.54 (11)	C5—C4—C3—C2	0.05 (18)
C7—C2—C3—C4	−0.87 (19)	C20—N1—C21—C22	103.43 (13)
C6—C7—C2—O1	−178.77 (11)	C21—N1—C20—O3	2.70 (18)
C6—C7—C2—C3	0.86 (17)	C21—N1—C20—C19	−178.39 (10)
C6—C8—C9—C10	140.17 (12)	C12—C11—C10—C9	−109.46 (13)
C18—C19—C20—O3	−5.24 (18)	C13—C14—C15—C16	−55.96 (13)
C18—C19—C20—N1	175.83 (11)	C13—C12—C11—C16	54.57 (13)
C18—C17—C8—C6	70.41 (12)	C13—C12—C11—C10	179.40 (10)
C18—C17—C8—C9	−163.77 (10)	C11—C16—C17—C18	−179.76 (9)
C19—C18—C17—C16	135.66 (13)	C11—C16—C17—C8	61.18 (12)
C19—C18—C17—C8	−104.64 (14)	C11—C16—C15—C14	52.15 (13)
C16—C17—C8—C6	−169.12 (9)	C11—C12—C13—C14	−56.40 (13)
C16—C17—C8—C9	−43.30 (12)	C23—C22—C21—N1	165.79 (11)
C16—C11—C10—C9	16.46 (16)	C15—C14—C13—C12	56.89 (13)
C17—C18—C19—C20	173.97 (11)	C15—C16—C17—C18	−53.39 (13)
C17—C16—C11—C12	77.81 (12)	C15—C16—C17—C8	−172.45 (9)
C17—C16—C11—C10	−46.76 (13)	C15—C16—C11—C12	−51.11 (13)
C17—C16—C15—C14	−75.12 (13)	C15—C16—C11—C10	−175.68 (10)
C17—C8—C9—C10	14.53 (16)	C1—O1—C2—C7	−178.70 (12)
C2—O1—C1—O2	−2.74 (12)	C1—O1—C2—C3	1.63 (13)
C2—C7—C6—C8	−178.69 (10)	C1—O2—C3—C4	179.60 (12)
C2—C7—C6—C5	−0.07 (17)	C1—O2—C3—C2	−1.84 (13)
C8—C6—C5—C4	177.87 (11)	C24—C22—C21—N1	−70.39 (13)
C8—C9—C10—C11	−0.56 (19)		

### Hydrogen-bond geometry (Å, º)

Cg2 is the centroid of the benzene ring C2–C7.

**Table:**

*D*—H···*A*	*D*—H	H···*A*	*D*···*A*	*D*—H···*A*
N1—H1···O3^i^	0.896 (16)	2.105 (16)	2.8938 (13)	146.3 (13)
C7—H7···O1^ii^	0.984 (16)	2.503 (16)	3.4264 (15)	156.3 (12)
C1—H1*B*···*Cg*2^i^	0.978 (16)	2.595 (15)	3.4578 (12)	147.4 (11)

Symmetry codes: (i) *x*−1, *y*, *z*; (ii) −*x*+1, −*y*+2, −*z*+1.
