# Supplementary figures and images for: Crystal structure of obscurine: a natural product isolated from the stem bark of B. obscura
Source: Acta Crystallogr E Crystallogr Commun. 2015 Jun 10;71(Pt 7):o457–8. doi: 10.1107/S2056989015010567 (PMC4518939; doi:10.1107/S2056989015010567)

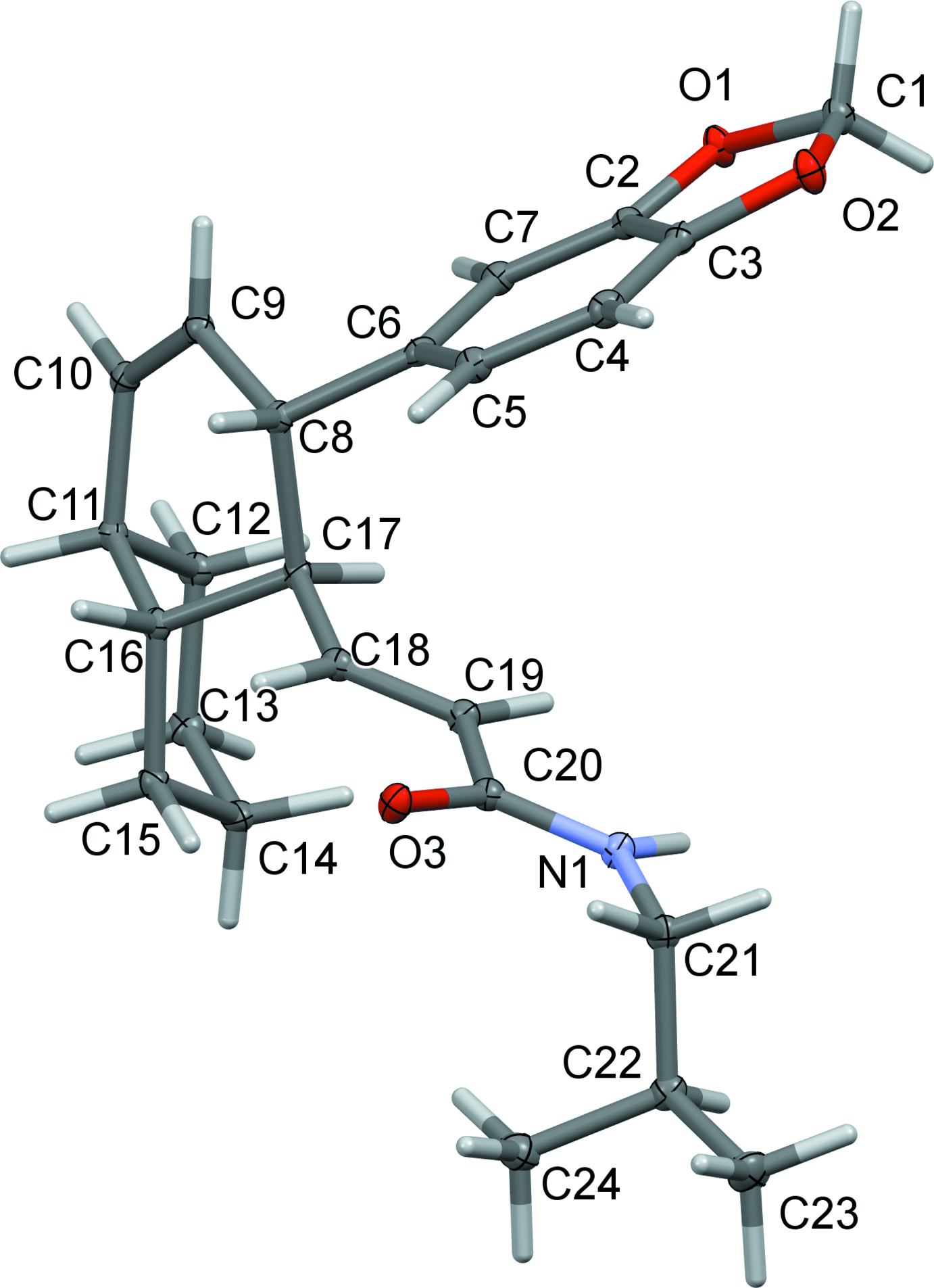

Supplement: Supplementary file 3 [file e-71-0o457-fig1.tif]

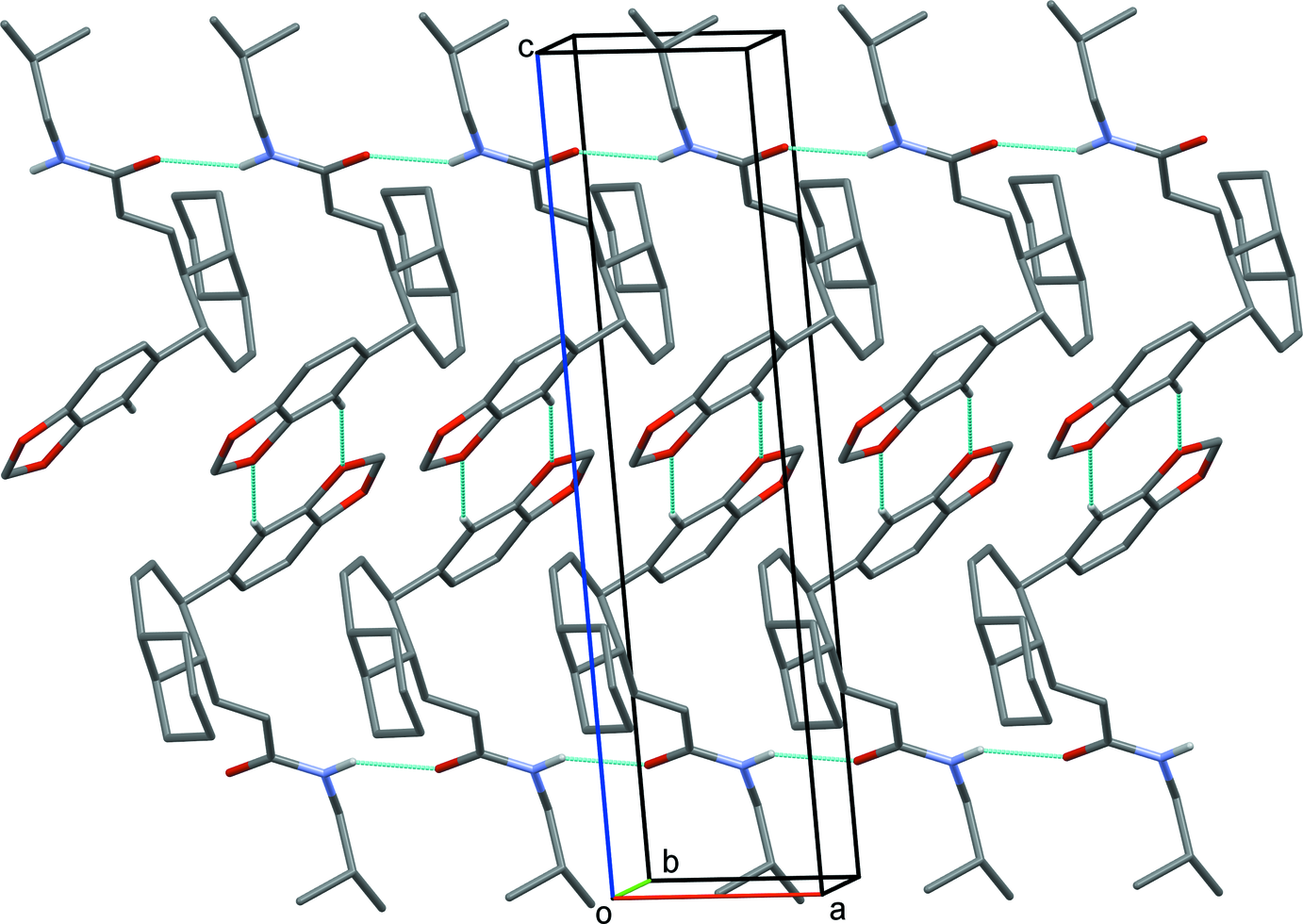

Supplement: Supplementary file 4 [file e-71-0o457-fig2.tif]
